# Health Promotion in European Higher Education Institutions: An Integrative Literature Review

**DOI:** 10.3389/phrs.2025.1608126

**Published:** 2025-12-09

**Authors:** Mariana da Silva de Lima, Jorge Manuel dos Santos Conde, Jorge Manuel Rodrigues Bonito

**Affiliations:** 1 Doctoral Program in Educational Sciences at the University of Évora, Évora, Portugal; 2 Federal Institute of Education, Science and Technology of Ceará, Ceará, Brazil; 3 Coimbra School of Health Technology - Polytechnic Institute of Coimbra - IPC, Coimbra, Portugal; 4 Center for Research in Education and Psychology of the University of Évora (CIEP-UE), Évora, Portugal; 5 Research Center in Didactics and Technology in Teacher Training of the University of Aveiro, Aveiro, Portugal

**Keywords:** colleges, health promotion, health-promoting universities, healthy universities, higher education

## Abstract

**Objectives:**

This research aimed to determine the state of the art of health promotion actions in higher education institutions of European Union member countries.

**Methods:**

To achieve the proposed objective, an integrative literature review was conducted. The collection of analyzed articles was carried out across four databases and a data compilation, where studies published between 2016 and 2022 were reviewed. The analysis was conducted through the description of the actions in health promotion (HP).

**Results:**

Fourteen articles were selected for analysis. The HP actions described primarily target students. Some of the main topics covered in the studies are health habits related to gender differences, recreational substances and health education.

**Conclusion:**

Although research in this area is increasing, it appears necessary to further disseminate the principles of the health promotion universities movement to raise awareness across the entire academic community. This could more easily lead to the development of more effective HP actions directed at the entire academic community and those outside the institutions.

## Introduction

The concept of health promotion (HP) is related to “a decent standard of living, good labor conditions, education, physical culture, means of rest and recreation.” [1].

Spaces such as workplaces, institutions that provide health services, as well as educational environments like schools and universities, have great potential for implementing actions and projects in HP. The way these locations are structured and organized allows for the intersectoral coordination necessary to achieve the objectives of an HP intervention [2]. Among the sectors mentioned, higher education institutions (HEIs) play an important role in our society. These institutions are responsible for preparing future professionals, individuals who will occupy decision-making positions in the future and manage key sectors [3]. Thus, HEIs have come to be seen as extremely promising contexts for developing health-promoting spaces, both for their educational community and the communities they are part of [4].

From there, the movement of Health Promoting Universities (HPUs) began to gain momentum and followers, leading to its official establishment in 1996 through the First International Conference on Health Promoting Universities, hosted by the University of Lancaster in England [5]. Building a health-promoting HEI involves developing a health culture by integrating institutional processes and policies. The institution should aim to empower its target audience, create actions in a participatory manner with all sectors, and consider goals to be achieved concerning equity, environmental sustainability, and health-friendly work conditions [6].

Regarding the various guiding documents for HPUs, the Okanagan Charter [7], published in 2015, serves as a guide for the development of HP actions and policies in HEIs. This document, published following the International Conference on Health Promoting Universities & Colleges, serves as an inspiration for institutions and mandates that HP be developed holistically, emphasizing intersectoral collaboration and adhering to certain guiding principles. These principles aim to create an HEI that reflects an educational and work environment promoting health for its academic community and surrounding community [8]. Thus, the analysis of actions by HEIs aligned with the HPU movement finds in the Okanagan Charter an appropriate guiding document for evaluating their HP policies and interventions.

In recent years, the European Union has witnessed the spread of the HPU movement and the multiplication of various HPU networks within its territory, such as the German Network of Health Promoting Universities, the UK National Healthy Universities Network, and the Spanish Network of Healthy Universities [9].

At this point, it seems important to take stock, based on scientific literature, of the HP actions of European Union HEIs, characterizing them through a descriptive analysis and supplementarily through the Okanagan Chater. The characterization refers to the target audience of the action, the objectives of the action and the stages of implementation of the interventions.

## Methods

To characterize the interventions and actions in HP at European HEIs, an integrative literature review was conducted [10], encompassing the following steps: formulation of the guiding research question, establishment of criteria for the inclusion and exclusion of documents to be analyzed, determination of the information to be extracted from the selected documents, conducting a critical analysis of the selected documents, and finally, discussion, synthesis, and presentation of the results.

To formulate the guiding research question for the integrative review, the PICO strategy was employed [11]. The population comprised the academic community of European Union higher education institutions (HEIs). The intervention was related to HP in these institutions, with comparison made through the articles included in the integrative literature review. The outcome refers to the characterization of HP actions in the chosen population. Both quantitative, qualitative, and mixed-method studies were selected. Based on these elements, the guiding question was formulated as follows: “What are the characteristics of health promotion interventions related to the health-promoting universities network movement in European Union?”

Document selection was conducted in April 2023 through online access to reference databases in the fields of health and education: Biblioteca Virtual em Saúde (BVS–Virtual Health Library), Education Research Information Center (ERIC), PubMed, Scopus, and Web of Science. The keywords “Health promoting universities,” “Healthy universities,” and “Health promoting universities network” were combined with the keywords “students” and “education” using Boolean operators OR and AND.

“Health promoting universities” OR “Healthy universities” OR “Health promoting universities network” AND “students” AND “education” was the *string* used. It was specified in the databases that the terms should appear in the title, abstract, or keywords of the articles.

Inclusion criteria for the articles were as follows: documents in Portuguese, English, or Spanish that addressed the research question previously established for the study. Additionally, publications considered for the research were complete articles published in scientific journals that either focused on the topic of health promotion in HEIs or utilized the health-promoting universities (HPUs) model as the guiding concept of the study. Finally, we chose the articles using the following quality criteria: citation of the article by other authors, clearly defined objectives, presence of consistent methodology, conclusions in accordance with the objectives and conclusions supported by the results. The selected studies for analysis were also subject to a publication date criterion, with only studies published from 2016 to 2022 being analyzed, given that the Okanagan Charter, the document used for study analysis, was published in 2015.

**FIGURE 1:**
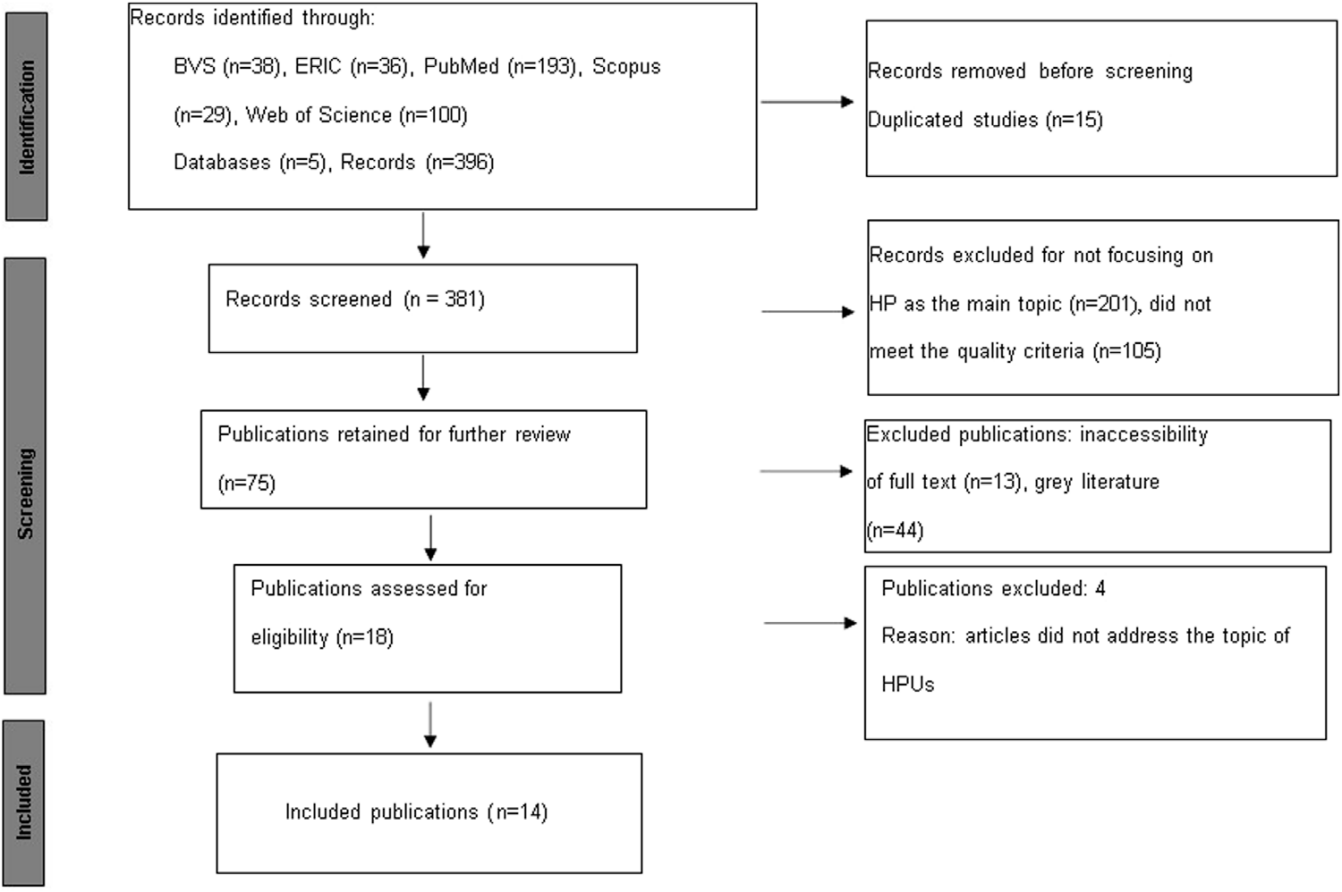
Flowchart of the Search Strategy in Databases (Integrative review, countries belonging to the European Union, 2014–2023).

A total of 396 articles were found in the initial search. The list of documents was exported from each database in.csv format for subsequent upload into the free software Ryyan. The articles were analyzed and selected according to the established criteria. Figure 1 details the flowchart followed in the process of obtaining the analyzed documents.

The analysis of all the articles was conducted through the description of the actions in HP and supplementarily according to the principles of the HPU movement, using the criteria established in the “Okanagan Charter”, specifically ensuring approaches encompass all university functions, developing transdisciplinary and intersectoral collaboration, valuing local communities and taking responsibility for current universal needs, particularly in the area of environmental health. The results of the health promotion actions were analyzed considering their scope within the institution and their impact on the academic community.

To aid in the study of the documents, a table was created outlining the key components of each investigation. Table 1 presents specific elements of each document.

**TABLE 1:** Summary of selected articles from online databases. (Integrative review, countries belonging to the European Union, 2014–2023).

Reference number	Authors/Year	Country	Objectives	Participants	Actions
E1	Newton et al. [12]	England	Study how the concept of “healthy university” is operationalized in two British universities	Students and staff at two British universities	Document analysis Field observation Semi-structured interviews with students and staff
E2	Holt and Powell [13]	England	To examine the health behaviors of students at a British university	University students at a British university	Preparation and application of a structured online questionnaire
E3	Haas et al. [14]	England	Analyze the extent to which university students are seeking to improve their health habits	University students from two London universities	Application of a structured online questionnaire on changes in health habits
E4	Bickerdike et al. [15]	Ireland	To examine differences in health behavior between the sexes and identify positive predictors of mental health in an Irish HEI	University students at an Irish university	Application of a structured online questionnaire on health habits
E5	Jack et al. [16]	England	To study how well a London HEI is meeting the psychosocial wellbeing needs of immigrant students	Immigrant university students at a London university	Application of narrative research, photovoice and interviews with key informants
E6	Martínez García and Zambrano Alvarez [17]	Spain	Describe the HP actions carried out at the University	Academic community of the Pablo de Olavide University and society in general in the Andalusia region	Events: World Health Day, World AIDS Day Training lectures: Eating habits, Health, society and education, affective-sexual education, meditation, eating disorders. Round tables with professionals on youth and health, mediterranean nutrition and therapy Training of health multipliers in the youth group to replicate knowledge among youth inside and outside the university. Alternative campaigns created by students
E7	Bachet et al. [18]	Germany	To research the level of collaboration between key actors to promote the health of the academic community through network analysis at a German university	Those responsible for the sectors that contribute to HP at the university under study	Identification of actors in the HEIs Application of a semi-structured questionnaire Data analysis using the SPSS software
E8	Limuratti et al. [19]	Austria	Demonstrate the effectiveness of a HP program entitled “healthy study start”	University students at an Austrian university	Survey of students’ needs through a questionnaire Development and implementation of a PdS program called “healthy study start” where the notion of coherence, sociability and notions of collective work are worked on Testing the effectiveness of the program through surveys with students participating in the program
E9	Reichel et al. [20]	Germany	Exemplify how to conduct effective health research with university audiences Provide a platform for discussions and suggestions for future investigations	Students at the University of Mainz, Germany	Aplicação de questionário e análise dos dados levantados
E10	Ahlstrand et al. [21]	Sweden	To describe HP resources and health-related factors in a group of students from universities in southern Sweden	First-year higher education students studying health and social services	Distribution of questionnaires among the target audience and analysis of the data collected
E11	Brandão et al. [22]	Portugal	Describe the development of an e-health monitoring program entitled ‘integrated e-health monitoring system for health management in Universities (e.cuidHaMUs™). The program aims to detect risk behaviors related to non-communicable diseases and implement problem-solving measures	Professionals working at the university of Aveiro, Portugal	Presentation of the design of the e.cuidHaMUs™ program. Development of an eHealth web platform to share information between different stakeholders. Application of a questionnaire to assess the health status of workers at higher education institutions (e.cuidHaMUs.QueST®)
E12	Gonçalves and Romero-Rodríguez [23]	Portugal and Spain	To evaluate the knowledge and practices of cooks associated with salt in public universities in the north of Portugal and Spain	Food handlers working in university canteens in northern Portugal and Spain	Application of a questionnaire among food handlers and analysis of the data collected
E13	Sanchis-Soler et al. [24]	Spain	To analyze the effects of a training program on the level of physical activity, mental health and body composition in sedentary university students	University students, self-declared sedentary, from a Spanish university	Submission of 14 students to a physical activity training program developed for the research in question Application of a questionnaire to measure sleep quality and aspects related to mental health before and after the training program Anthropometric measurements before and after the training program
E14	Teixeira-da-Costa, et al. [25]	Portugal	Identify and describe practices related to the consumption of tobacco products and their distribution according to gender among students at a Portuguese university	University students from the university of Algarve	Application of a questionnaire on tobacco consumption and analysis of the data collected

## Results and Discussion

Regarding the methodological approach used, 12 articles employed quantitative methodology, while two used qualitative methods (E1 and E5). Cross-sectional design was the most used type in the selected studies, with research E6, E8, and E13 utilizing longitudinal design. Based on the analysis of the selected studies, two categories were created according to the approach used in the study. The first category refers to studies aimed at measuring some aspect related to HP. These are diagnostic studies, including behavioral, educational, epidemiological, administrative, or organizational diagnostics. The second category pertains to studies that describe HP practices in HEIs. A larger number of the analyzed investigations belong to the first group.

### Diagnostic Research

#### Diagnostic Studies in the Student Population

These investigations specifically focus on the general use of healthcare services, dietary behaviors, alcohol consumption, smoking, sexual health, mental health, and the use of illicit psychoactive substances (E2, E3, E4, E10, E13, and E14). The studies aim to collect data for a future health promotion (HP) action plan directed at university students. Study E9 also belongs to this subgroup; however, the authors focus on analyzing the data collection instrument, as described further below.

In E2, the researchers distributed a 60-item questionnaire via institutional email to collect data on students’ health behaviors. In this study, the questionnaire was developed by the research team and made available online through the university’s email to all students attending the campus. To ensure the success of the research, the university’s service department collaborated closely with the researchers. As a result, the investigators observed, among other data, a higher prevalence of tobacco use among male students.

In E3, while investigating changes in health habits among university students in London, the researchers collaborated with student associations to distribute the data collection instrument. Support from the student sector contributed to a sense of value for this group within the university, as well as making the research instrument more widely accepted. It was concluded that university students at the analyzed HEIs have healthier habits compared to those reported in previous studies, suggesting an improvement in healthcare among the student population.

Within the group of diagnostic studies, in study E4, Irish researchers analyzed health behaviors and lifestyles of university students, including differences in lifestyles between men and women. To this end, a questionnaire was also developed and made available to all enrolled students at the studied HEI. The researchers found discrepancies in men and women regarding their actual weight and overweight status, with this discrepancy being more significant among men. This result is presumed to be related to aesthetic pressures directed towards the female population, in addition to the already documented finding that women are more concerned with health issues. Furthermore, the study also observed a greater tendency among women towards healthier eating, lower involvement in excessive alcohol use and recreational substances, and fewer sexual partners over their lifetime. However, female students reported higher stress levels and lower tendencies to remain physically active compared to men.

In study E10, the researchers examined the lifestyle and health resources of university students from six Swedish HEIs, specifically from health and social care programs. The aim was to describe the associations between health, perceived wellbeing, and personal health-promoting resources. The data revealed that students had good health and wellbeing, with low levels of alcohol and recreational substance use, good dietary habits, regular physical activity, and a strong perception and knowledge of personal health promotion. However, the data indicated that 40% of students had difficulties maintaining good sleep habits.

Regarding physical activity and wellbeing, study E13 stands out for analyzing a physical activity program and its outcomes. It serves as a test for developing a practical HP intervention. In this study, the researchers observed the effects of a physical training program on sedentary students at a Spanish university. This longitudinal study analyzed whether participation in the program influenced factors related to health and wellbeing among the participants. At the end of the study, improvements were observed in sleep quality, anxiety, and depression after 5 weeks of training, although the improvement in stress levels was not statistically significant.

In study E14, although it is also diagnostic research, it is part of an established program at the University of Algarve aimed at combating smoking. This intervention consists of institutional actions to raise awareness within the academic community about the harmful effects of smoking and focuses on the smoking cessation process. The program is developed in partnership with the Algarve Regional Health Administration and a food industry company. The study involves data collection to plan the continuation of interventions based on scientific evidence. A questionnaire was administered to the student body regarding their smoking habits. The results showed that among smokers, 45% reported smoking daily while 55% considered themselves occasional smokers, with no significant difference between men and women. The data also indicate that female smokers prefer conventional cigarettes, whereas men consume more tobacco/nicotine products. These findings may provide guidance for future HP actions for the studied population.

Regarding diagnostic research focused on the student population, study E9 describes the authors’ successful experiences in conducting health research within HEIs. The goal of the research was to foster a discussion platform among researchers, which aligns with one of the aspects of the HPU model: mutual collaboration in developing actions. In this study, the investigators reported their experience with data collection interviews in the university population and found that women, in general, are more inclined to respond to questionnaires than men. This finding suggests the need for efforts to encourage greater participation from men in future research.

Analyzing the topics more broadly, it is evident that most actions focused on the perception of gender differences in maintaining healthy habits (28.6%). The studies analyzed concluded that the female population pays more attention to health. Sleep quality and smoking were the subjects of 21.4% of the studies, while alcohol and recreational substances were the focus of 14.3% of the investigated studies. Table 2 presents the main topics worked in HP actions.

**TABLE 2:** Topics covered in HP actions. (Integrative review, countries belonging to the European Union and England, 2014–2023).

Types of researches	Topics	f (%)	Studies
Diagnostic studies in the student population	Gender differences in maintaining healthy habits	4 (28.6)	E2, E3, E4, E9, E14
	Smoking	3 (21.4)	E2, E3, E14
	Alcohol and recreacional substances	2 (14.3)	E3, E4
	Sleep quality	2 (14.3)	E10, E13
	Overweight	1 (7)	E4
Institutional diagnostic studies	Health promotion concept	1 (7)	E1
	Offer of health services	1 (7)	E5
	Mental Health	1 (7)	E5
	Healthy HEIs network	1 (7)	E7
	Nutrition	1 (7)	E12
Research based on reports of Health promotion actions	Health education	2 (14.3)	E6, E8
	Interinstitutional partnerships	1 (7)	E6
	Worker health	1 (7)	E11
	Psychosocial health	1 (7)	E8

f = Absolute frequency and percentage.

#### Institutional Diagnostic Studies

These investigations are focused on HEIs, their leaders and how they perceive PdS and how the different sectors act or organize themselves to promote health in the institution (E1, E5, E7 and E12). It is also interesting to highlight that E1 and E5 are the studies in our sample with a qualitative approach. E1 investigated how the concept of a healthy university is operationalized in a HEI that has a salutogenic approach and another university that does not have this approach in its organizational policy. It is an “instrumental case study” type study, in which an “exemplary case” and an “opposite case” were analyzed. At the university with a salutogenic approach, one of the main characteristics observed was the notion that the health and wellbeing of the academic community impact the development of the institution, both being interconnected. Thus, a strong tendency to value the individual was observed in this HEI, with the development of democratic processes for building actions and efficient channels of communication between students, employees and managers so that everyone could feel that their voice is valued and considered in the preparation of institutional policies and actions.

Study E5 deals with qualitative interpretative research. Although part of the research focused on data mining among the immigrant university public, we observed that the main focus was the perception of this public and the directors of the HEI studied about the health and wellbeing sector that serves the student population. The article also analyzed the organizational barriers perceived by immigrant students that impede their academic success. Using photovoice methodology, combined with narrative interpretation, ten refugee university students report aspects that influenced their mental health and wellbeing, including elements related to the university where they study. Semi-structured interviews were carried out with professionals and academics who could somehow influence the health and wellbeing of refugee students. The results reveal that these students have some difficulty accessing the HEI’s health and wellbeing services. According to the research, this fact is the result of several factors, such as lack of knowledge about the service offered, the fear of stigma for seeking help and the discomfort of talking to a stranger about “their personal stories”.

Finally, some students report difficulties in developing trust in the university’s health and wellness sector, arguing that there is a lack of diversity among the professionals in the sector, with the majority being Caucasian. From the students’ perspective, the lack of diversity among the staff hinders the development of empathy for their life stories and the quality of care. Key informants, in turn, confirm the students’ lack of awareness of the service, as well as recognizing structural and resource deficiencies in the health and wellness sector. Both groups contributed suggestions for possible changes and improvements to the sector, such as increasing awareness of the service’s existence and enhancing the representativeness of the professionals who serve the students, in addition to training them in managing typical traumatic situations faced by refugee students.

Study E7 analyzed the HPUs network in Germany, identifying key agents within institutions and the extent to which there was coordination to achieve the goal of promoting health within the academic community and its surroundings. The study focused on identifying key agents in the network and analyzed the level of interaction between these individuals. The research was conducted via a questionnaire distributed to 33 selected participants, representatives of 33 organizational units that are part of the network. The results show good interaction among network agents, with effective information flow and close communication levels between the analyzed network agents. It was also noted that, among the health promotion topics each agent considers important for their professional quality, physical activity and relaxation were among the most frequently mentioned. This suggests that these topics are valued in institutions that adhere to health promotion practices.

Concluding the analysis of diagnostic study articles, E12 examined the food services sector in two universities, one located in northern Portugal and the other in Spain. The food handlers responsible for meals in the cafeterias and restaurants of these institutions were subjected to a questionnaire. The aim was to understand how they manage salt in the food prepared for the academic community. The results showed that food handlers perceive that the population in both countries consumes excessive salt and that there is a real need to reduce salt in the meals prepared in higher education institutions. However, the study reports difficulties in reducing salt use in prepared food, citing consumer opinions and the fear of losing customers due to the belief that the food will be less flavorful. These results may assist in the development of interventions in the field of nutritional education for the university community.

### Research Based on Reports of Health Promotion Actions

We classify the second group of studies as “studies describing actions in HP” based on Suàrez-Reyes and Van den Broucke [26]. In their study, they classify this type of study as “intervention studies.”

The articles belonging to the second category (E6, E8, and E11) describe actions or programs related to HPU.

E6 outlines the trajectory of the Andalusian Network of Healthy Universities (RAUS) and its health promotion activities at Pablo de Olavide University. It details various partnerships, including those with the Regional Ministry of Health, the Andalusian regional government, and UNESCO, as well as other institutions such as hospitals, banks, and health-related companies. The article also reports on several events organized for the student body, university professionals, and the external community, including World Health Day, World AIDS Day, lectures on dietary habits, health, society and education, affective-sexual education, meditation, and eating disorders.

Additionally, roundtables were conducted with professionals on topics such as youth and health, Mediterranean diet, and therapy. Health multiplier training sessions were organized to disseminate knowledge among the youth both within and outside the university. The study also reports on alternative health and wellness campaigns developed by students. The RAUS exhibits actions that are closely aligned with the HPU movement. It is evident that various sectors within the institutions are involved in the development of these actions and the democratic decision-making process. The article reflects on the significance of universities embracing their social role in producing and disseminating scientific knowledge for the health and wellbeing of the general population and underscores the importance of the HPU movement.

Article E11 is one of the studies that describes a practical action in health promotion and is the only one in the sample that specifically targets staff within higher education institutions (HEIs). The aim of the work was to outline the research phase and the implementation of an occupational health plan at a Portuguese university. The University of Aveiro serves as the incubator for this program, but the proposal is to adapt and apply the experience to any type of organization.

The plan consists of the implementation of the “Integrated Electronic Health Monitoring System for Health Management in Universities” (e.cuidHaMUsTM). The proposal is based on three phases: awareness, motivation, and action. The program focuses on two main functions. The first is health monitoring through screening and prospective surveillance via an online platform. The second function involves intervention in avoidable non-communicable diseases through individual health records of workers. A pilot test was conducted using a short questionnaire administered to workers who were called to the university’s health department for their annual medical check-up, achieving a 93% participation rate among the targeted staff.

The data revealed a low level of physical activity and issues with non-communicable diseases. The results are intended to assist in building a culture of health promotion in the context of occupational health within HEIs. This way, health data of a specific organizational group can inform decision-making sectors both within and outside the institution, facilitating evidence-based actions and public policies. Simultaneously, the program is a collaborative approach to health promotion, being self-administered and optional.

Concluding the category of practical actions in health promotion, Article E8 describes an initiative at an Austrian university that focuses on social skills and the sense of coherence related to the health of first-year students in health and social care courses. The study details important stages of health promotion interventions, such as the data collection phase conducted collaboratively with students through questionnaires and “Open Space” sessions, where students reported factors influencing their health during their first year of higher education.

The study also presents the implementation phase of the program developed from the collected data, titled “Healthy Study Start,” aimed at first-year students. This program includes informational sessions on health, wellbeing, and social skills, interactive activities among students, and teamwork exercises outdoors. It is important to note that this study is the only one that addressed students with physical disabilities, mentioning that the program and its evaluation are adapted to support inclusion. Finally, the article describes the evaluation of the intervention by the target audience. The results indicate that the action improved management skills, social support awareness, friendliness, and effectiveness related to teamwork among participants. This suggests that health promotion actions at the start of university life can support the development of social qualities, which may contribute to the promotion of student wellbeing throughout their academic journey.

In the present integrative literature review, a higher number of diagnostic studies in HP were observed in the scientific literature, aimed at developing actions based on perceived needs (articles E1, E2, E3, E4, E5, E7, E8, E10, E12, E13, and E14). These results corroborate what was proposed in the Okanagan Charter, which states that HP research is one of the listed actions most related to the university [7]. In our study noting that there is significantly more published information in the scientific literature from diagnostic studies, whether targeting specific audiences or institutional, than in descriptions of proposed interventions or actions that have already been concretely implemented. This is related to the fact that research is one of the missions of universities, which means that these institutions already have resources and qualified professionals for this purpose.

Ferreira et al. [27], in their integrative review of articles describing HP actions in HPUs, found various concrete actions described in 17 selected articles. However, their research was not restricted to the European Union (EU), but analyzed universities across different continents. The present literature review focused on HEIs in the EU, as it was understood that the countries in this continent share certain cultural and social context similarities. Additionally, it is important to note that the selected articles were published from 2016 onwards. Therefore, even though England is no longer part of the EU, it was deemed appropriate to include it in our sample, given that their exit from the European bloc was only finalized in 2020.

The reason for the chronological criterion is that the Okanagan Charter, which is our guiding document for analysis, was published in 2015. This document provides a guide for developing health promotion interventions in universities according to the principles of the HPU movement. One of the key principles is the promotion of research to enhance knowledge about the health of the university community. This is essential for developing evidence-based actions and projects. In this context, diagnostic studies align with this principle; however, it is important to emphasize that the diversity of the audience needs to be addressed.

One of the premises of health promotion in HEIs is the focus on all sectors of the HEI, including students, professionals, and the external public [28]. In the present literature review, it was observed that most investigations focus exclusively on the student population (E2, E3, E4, E8, E13, and E14), comprising 50% of the analyzed studies. This indicates a gap in research, whether diagnostic or experiential, that is directed towards professionals in health-promoting HEIs.

When referring to the community outside of institutions, this gap is even more pronounced. Study E10 was the only one that focused exclusively on the health of staff, as the presented health monitoring program initially targeted HEI professionals. While directing actions toward students is fundamental, it is important to emphasize that the health of staff and the external community of HEIs are also central to the HPU movement [29]. According to Arroyo [30], HPUs should be committed to developing a culture of health promotion within the entire academic community as well as in the external community, that is, in society at large. Society and universities are closely linked, and the knowledge produced within universities should find ways to reach the surrounding community [31].

The concern for environmental quality is also highlighted in E6. The research discusses the need for an approach that promotes environmental health and sustainability in spaces. The dimension of environmental health in health-promoting spaces, as this aspect can positively influence individuals’ daily lives [32].

Hof-Nahor and Biswas [33] report how a university in Israel has increased the use of green spaces for hosting events with students and staff, with the aim of fostering a culture that values environmental quality within the academic community. It is well established that green spaces contribute to the wellbeing of individuals who frequent these areas [34]. Evidence indicates that vegetated areas have the potential to reduce stress, promote an effective learning environment, and enhance cognition among students [35]. The Okanagan Charter includes as one of its principles the need to integrate health, wellbeing, and sustainable development. Furthermore, according to Arroyo [16], “HPUs have the mission to advocate for health using the conceptual/operational framework of social determinants of health and the Sustainable Development Goals (SDGs).”

Finally, E8 addresses the aspect of social relations and cooperation within the academic sphere, which are important elements for health and wellbeing. The study engages with the concept of “sense of coherence” developed by Antonovsky, who asserts that health is achieved through individuals’ personal resources, including psychosocial elements [36]. Social competencies, as well as a sense of empathy and collaboration, should be strengthened among individuals. These factors are crucial for maintaining psychosocial health. The approach implemented in the study facilitates interaction among students, enabling the development of connections between them, which fosters a sense of belonging to the group, improving partnership and solidarity. These elements contribute to developing an identification with the university, which undoubtedly enhances a socially healthier academic environment [37].

Human beings are inherently social and social skills are crucial for their personal and professional development. The Okanagan Charter includes as one of its principles the establishment of interpersonal and intersectoral connections for creating healthy environments, highlighting the importance of this issue for people’s wellbeing. It is essential that health be viewed beyond the health-disease binary, and HP actions should also consider a wide range of issues to address the entirety of an individual’s life.

HEIs have increasingly been established as favorable environments for HP in various countries. Although research in this area is expanding, it is necessary for the principles of the HPU movement to be disseminated widely, becoming familiar to all stakeholders. This can more easily lead to the development of more effective HP actions targeted at the entire academic community and those outside the institutions. It is also essential to develop and strengthen actions and research concerning health and environmental sustainability, as well as psychosocial health and wellbeing within the framework of HPUs.
