# Supplementary material for: Health Promotion in European Higher Education Institutions: An Integrative Literature Review
Source: Public Health Rev. 2025 Dec 9;46:1608126. doi: 10.3389/phrs.2025.1608126 (PMC12722967; doi:10.3389/phrs.2025.1608126)
Supplement: Supplementary file 1 [file Table1.doc]

| *Documents found in initial search by database (Integrative review, countries belonging to the European Union, 2014-2023).* | | | |
| --- | --- | --- | --- |
| **Database** | **Search options** | **Search equation** | **Number of Documents Found** |
| BVS | Publications from 2016 to 2022  Keywords in Title, Abstract, and Subject  Peer-Reviewed | “Health promoting universities” OR “Healthy universities” OR “Health promoting universities network” AND “students” AND “education” | 38 |
| ERIC | Publications from the Last 10 Years  Peer-Reviewed  Publication Type: Articles  Descriptors Applied: Higher Education and Universities  Educational Level: Higher Education | “Health promoting universities” OR “Healthy universities” OR “Health promoting universities network” AND “students” AND “education” | 36 |
| PubMed | Publications from 2016 to 2022  Keywords in Title and Abstract  Articles in Spanish, English, and Portuguese  Peer-Reviewed | “Health promoting universities” OR “Healthy universities” OR “Health promoting universities network” AND “students” AND “education” | 193 |
| Scopus | Publications from 2016 to 2022  Limited to European Union countries  Articles in Spanish, English, and Portuguese | “Health promoting universities” OR “Healthy universities” OR “Health promoting universities network” AND “students” AND “education” | 29 |
| Web of Science | Publications from 2016 to 2022  Limited to European Union countries  Articles in Spanish, English, and Portuguese  Peer-reviewed  Category: Educational Research | “Health promoting universities” OR “Healthy universities” OR “Health promoting universities network” AND “ higher education students” AND “higher education institution” | 100 |
| ***Total*** | | | *396* |
